# The Significance of Hypophosphatemia in Deciding on an Optimal Clinical Choice of Parenteral Iron Therapy in Patients with Chronic Inflammatory Bowel Disease in Slovenia: An Umbrella Review and Economic Evaluation

**DOI:** 10.3390/healthcare14030393

**Published:** 2026-02-04

**Authors:** Rok Hren, Tamás Dóczi, Erika Országh, Tomaž Kocjan

**Affiliations:** 1 Syreon Research Institute, 1142 Budapest, Hungary; 2 Faculty of Mathematics and Physics, University of Ljubljana, 1000 Ljubljana, Slovenia; 3 Institute of Mathematics, Physics, and Mechanics, 1000 Ljubljana, Slovenia; 4 Department of Endocrinology, Diabetes and Metabolic Diseases, University Medical Centre Ljubljana, 1525 Ljubljana, Slovenia; 5 Department of Internal Medicine, Faculty of Medicine, University of Ljubljana, 1000 Ljubljana, Slovenia

**Keywords:** anemia, hypophosphatemia, ferric carboxymaltose, ferric derisomaltose, inflammatory bowel disease, pharmacoeconomic analysis

## Abstract

**Background/Objectives**: Iron-deficiency anemia (IDA) is a common extraintestinal complication of inflammatory bowel disease (IBD). Among high-dose intravenous (IV) iron options, ferric carboxymaltose (FCM) carries a higher risk of treatment-emergent hypophosphatemia than ferric derisomaltose (FDI), with potential clinical consequences. Slovenia’s healthcare setting, characterized by very low IV iron infusion tariffs and recent pricing in which FCM is substantially less expensive than FDI, warrants a setting-specific cost effectiveness evaluation. **Methods**: We integrated two methodological components: (i) a payer-perspective cost-effectiveness analysis using a patient-level microsimulation model with (ii) an umbrella review of systematic reviews and a targeted search of expert consensus statements on IV-iron-associated hypophosphatemia. **Results**: In the base case, FDI required fewer infusions than FCM (11.1 vs. 14.2 over 10 years) but generated only €95 in IV iron administration savings due to low tariffs, while drug procurement was €1166 higher with FDI than FCM. When incorporating the clinical impact of hypophosphatemia, incremental quality-adjusted life years (QALYs) were 0.136, yielding an incremental cost-effectiveness ratio (ICER) of €6590/QALY. The umbrella review consistently showed higher hypophosphatemia incidence with FCM (up to 92%) compared with other IV iron formulations (<10%), with recent recommendations emphasizing phosphate monitoring and risk mitigation through alternative formulations. **Conclusions**: Despite Slovenia’s low IV iron infusion tariffs and lower FCM price, FDI remained cost-effective in this model, largely due to its more favorable hypophosphatemia profile within the model. These findings suggest that hypophosphatemia risk should be considered when selecting IV iron therapy in routine IBD care.

## 1. Introduction

Anemia is a common extraintestinal complication of inflammatory bowel disease (IBD), with its main causes being iron deficiency due to chronic blood loss, dietary adaptations among patients with IBD, and chronic inflammation that reduces iron absorption from the gastrointestinal tract [1]. IBD-associated anemia has a significant impact on patients’ physical and cognitive functioning, increases hospitalization rates, and reduces quality of life [2,3,4,5,6]. The European Crohn’s and Colitis Organization (ECCO) recommends intravenous (IV) iron as the first-line treatment for, inter alia, patients with clinically active IBD and iron-deficiency anemia [4,5], since the absorption of oral iron is limited in patients with intestinal inflammation [7]. Historically, IV iron is considered a safe medication, with an estimated rate of serious adverse reactions of approximately one per 200,000 administrations [8,9].

Among the available high-dose IV iron preparations used for the treatment of iron deficiency, there are two commonly used and clinically comparable agents: ferric carboxymaltose (FCM) and ferric derisomaltose (FDI), previously known as iron ferric isomaltoside. Safety distinction in clinical practice is hypophosphatemia, which occurs frequently with FCM (in up to 92% of cases) and saccharated ferric oxide (SFO), but rarely with other iron preparations like ferumoxytol, low-molecular-weight iron dextran (LMWID), or FDI (in 2–8% of cases) [10,11,12,13,14,15,16,17,18]. The mechanism is not fully understood; however, it is most likely related to the increase in intact fibroblast growth factor 23 (FGF-23), a phosphatonin that enhances renal phosphate excretion [13,19,20,21,22] after use of FCM.

Hypophosphatemia [23,24] may in approximately 10% become severe (phosphate < 0.32 mmol/L or 1 mg/dL) [25], symptomatic, or persistent for up to six months, leading to disturbances in bone and mineral metabolism and the development of osteomalacia [26,27,28,29,30,31,32]; in one case, even burosumab, a therapeutic monoclonal antibody against FGF-23, was proposed as an off-label treatment option [33]. Severe hypophosphatemia has been documented after both single and repeated FCM infusions, particularly in individuals with normal renal function and high cumulative iron doses [27,34,35,36,37,38,39,40,41,42,43,44].

In recognition of these risks, the UK Medicines and Healthcare products Regulatory Agency (MHRA) issued a 2020 Drug Safety Update advising serum phosphate monitoring in patients receiving multiple or long-term FCM treatments or those with predisposing factors for hypophosphatemia such as IBD [22,45]. Early identification and management are crucial to prevent chronic complications like osteomalacia and fractures, including those requiring surgery [45]. The ECCO 2024 guidelines have also addressed the risks in relation to FCM use in IBD patients [5], and the Summary of Product Characteristics (SmPC) for FCM now includes a special subsection under Section 4.4 specifically related to hypophosphatemic osteomalacia.

Moreover, there is growing perception that differences in the incidence and consequences of hypophosphatemia between IV iron formulations may also have important economic implications. Several pharmacoeconomic cost–utility (CU) analyses conducted across diverse healthcare settings, including China [46,47], the United Kingdom [48], Norway [49], and Sweden [50], have consistently shown FDI to be the dominant strategy, being both more effective and less costly than FCM. However, these evaluations were performed for pricing conditions where FDI and FCM were of comparable cost, and where iron infusion expenses were relatively high compared to the drug cost itself.

In Slovenia, by contrast, the cost structure differs substantially; iron infusion costs are exceptionally low (under €40 per infusion), and as of summer 2025, the price of FCM has decreased to 42% of the cost of FDI. These two parameters together justify a new economic evaluation to determine the potential consequences of this significant pricing shift. Moreover, Slovenia provides a representative model for Central and Eastern European (CEE) healthcare systems [51,52,53,54], with a well-developed and transparent pharmaceutical framework [55].

The objectives of the present study are therefore twofold: (i) to extend previous pharmacoeconomic evaluations by incorporating the current Slovenian cost environment, and (ii) to place these economic findings within the context of the latest clinical evidence, including recent systematic reviews and expert consensus statements. In doing so, this study aims to provide a comprehensive assessment of both the economic and clinical implications of FCM and FDI in the management of iron deficiency anemia (IDA) among patients with IBD.

## 2. Materials and Methods

Our methodological approach integrated pharmacoeconomic modeling (Section 2.1) with an umbrella review synthesizing evidence from systematic reviews and targeted search of consensus statements (Section 2.2).

### 2.1. Pharmacoeconomic Modeling

#### 2.1.1. Model Structure

**Figure A1:**
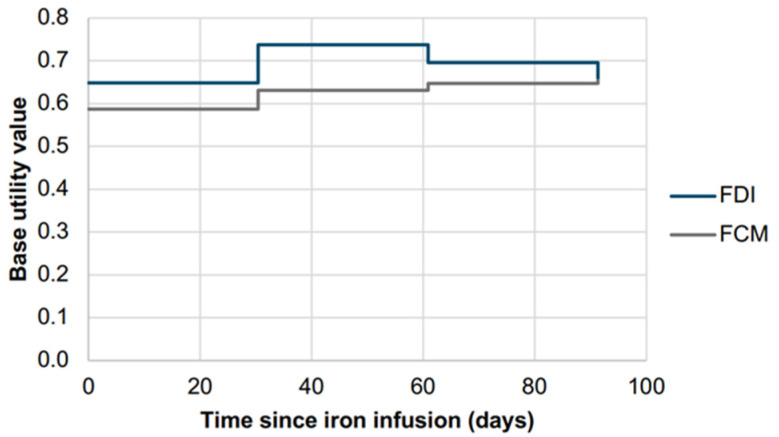
Baseline utility values derived from SFv36 data [48]. FDI—ferric derisomaltose; FCM—ferric carboxymaltose.

A cost-effectiveness analysis was conducted to evaluate FDI compared to FCM for patients with IDA and IBD in Slovenia. We conducted our analysis using the CU-IDA Model [48] to estimate the incremental costs and health benefits of a treatment of IDA in patients with IBD. The CU-IDA Model is a patient-level microsimulation model that simulates individual iron requirements and the number of infusions per treatment cycle according to the product characteristics of each IV iron formulation. Baseline characteristics were derived from the PHOSPHARE-IBD randomized controlled clinical trial (RCT), which enrolled patients from 20 outpatient hospital clinics across Austria, Denmark, Germany, Sweden, and the UK [12,38]. We used the CU-IDA Model as it was previously applied across multiple jurisdictions [46,47,48,49,50], while the PHOSPHARE-IBD study is pivotal, as it provided the first high-quality prospective evidence in an IBD population demonstrating that hypophosphatemia is a formulation-specific and clinically relevant adverse effect of IV iron therapy. The analysis assumed equivalent hematological responses and hemoglobin increases between FDI and FCM, consistent with findings from PHOSPHARE-IBD [12]. The key difference captured by the model related to the incidence of hypophosphatemia. Although other adverse events could have been considered, the analysis was deliberately conservative and focused solely on hypophosphatemia. For example, Pollock and Biggar [56] reported a lower risk of serious or severe hypersensitivity reactions with FDI compared with FCM and iron sucrose; however, this difference was not incorporated into the model. In the Slovenian adaptation of the model, as in prior versions [47,48,49,50], fractures were excluded to ensure a scientifically rigorous approach; while emerging data shows an increased fracture risk, no peer-reviewed data documenting the risk in clinical practice has been published so far. Data on quality-of-life differences between patients receiving FDI and FCM were based on the PHOSPHARE-IBD study [12,57], which assessed health status using the SF-36v2 questionnaire (36-Item Short Form, second version) [58]. This approach also allowed us to quantify the negative effects of symptomatic hypophosphatemia, such as general weakness and fatigue (baseline utility values for FDI and FCM can be found in Figure A1 of Appendix A [48]). The modeling structure, treatment sequencing, and decision rules followed those described previously [47,48,49,50], with all parameters, except those explicitly detailed below, remaining consistent with the reference model [48]; model parameters are summarized in Table 1. Simulations were performed from the payer perspective, including 2000 virtual patients with IBD treated for IDA. Indirect costs were excluded, consistent with Slovenian payer guidelines. Additional details of the health economic model are provided in Appendix A.

The analysis adhered to Slovenian pharmacoeconomic guidelines, applying a willingness-to-pay (WTP) threshold of €25,000 per QALY [61] and discount rate of 3.5% for both costs and outcomes. The incremental cost-effectiveness ratio (ICER) was calculated to assess the value of FDI relative to FCM over a 10-year time horizon.

#### 2.1.2. Payer Perspective Costing

Drug acquisition costs were derived from the most recent hospital procurement data and publicly available price listings. The cost for FDI was obtained from recent tenders in 2025 (€0.19 per mg), while the cost for FCM was based on the Slovenian official list price (€0.08 per mg). These unit costs were assumed to apply both in the initial treatment year and in subsequent years. Administration costs for iron infusions were obtained from the Slovenian payer tariff list for 2025, using the code APL022 (€35.06), which covers all non-pharmaceutical costs associated with iron infusion, including nursing time, consumables, patient observation, and proportional overheads such as utilities, facility maintenance, and capital depreciation.

The model also accounted for adverse events. For patients treated with FCM, the model assumed serum phosphate measurement after the first IV administration. If hypophosphatemia was detected, the patient would receive phosphate replacement therapy—either oral [22] or intravenous [62], depending on severity—and undergo an additional follow-up measurement. If a second iron infusion was required, phosphate levels were again checked afterward. For the costs of IV phosphate administration, we used the tariff APL005 of €134.78 from the Slovenian payer price list for 2025. The cost of serum phosphate measurement was calculated based on the price of the test (€1.30) and the sample collection fee (€2.50), as specified in the Synlab laboratory price list (https://www.synevo.si/images/Medicover/Cenik_Synevo_Adria_lab.pdf; accessed on 28 January 2026). For patients receiving FDI, phosphate monitoring was not included in the model, as this requirement is absent from the product information, and the PHOSPHARE-IBD clinical trial reported no severe cases of hypophosphatemia [12].

#### 2.1.3. Uncertainty Analysis

To explore the impact of parameter uncertainty, we conducted probabilistic sensitivity analysis (PSA). In this approach, model input parameters were simultaneously varied within their assigned probability distributions, generating outcome distributions that reflect overall model uncertainty. The PSA comprised 1000 Monte Carlo simulations per virtual patient.

In addition to the base case scenario, which assumed a lower number of IV infusions for FDI compared with FCM (reflecting the maximum weekly cumulative dose of 1000 mg for FCM), a scenario analysis was conducted in which FCM was offered at zero cost.

As part of the sensitivity analysis, we also incorporated alternative IV iron administration costs based on Danish data. In Denmark, the cost of IV iron administration in 2023 was 1529 DKK (drop from 2855 DKK in 2018 [63]), which, after adjustment for gross domestic product in purchasing power parity and conversion to euros at the prevailing exchange rate, corresponded to €146.80 (compared with €35.06 in the base case scenario). The purpose of this particular scenario was to assess cost-effectiveness under conditions reflecting IV iron administration costs typical of developed healthcare systems; for reference, the corresponding infusion cost in another high-income setting was 3656 NOK (around €310) in Norway [49]. Denmark was selected because, similar to Slovenia, IV iron therapy is reimbursed using a split tariff structure, with the cost of administration and the cost of the iron product reimbursed separately. In contrast, in countries such as the United Kingdom, IV iron therapy is reimbursed as a bundled, per-infusion price [48].

### 2.2. Umbrella Review of Systematic Literature Reviews and Targeted Search of Consensus Statements

A comprehensive literature search was conducted on 1 September 2025 using PubMed and EMBASE, with no limitations on publication date and with restrictions on the publication/study type. The umbrella literature review was conducted and reported in compliance with the PRISMA 2020 Statement [64]. Any peer-reviewed systematic review or meta-analysis reporting on hypophosphatemia during IV iron treatment of IDA were eligible. Duplicate records were removed both within and across databases. When identical work was published first in conference proceedings and later in a peer-reviewed journal, the proceedings version was treated as a secondary report and discarded. We adopted the format of a narrative review. Records that did not meet any exclusion criteria at the full-text screening stage were included. A more detailed description of the methodology, including PICOS, search strings, and exclusion criteria during title/abstract and full-text screening, is reported in Appendix B. In addition to the umbrella review, a targeted search was conducted to identify recommendations and consensus statements on hypophosphatemia during IV iron treatment of IDA.

## 3. Results

### 3.1. Pharmacoeconomic Modeling

Table 2 summarizes the results of the cost-effectiveness analysis, showing that in the base-case scenario for treating IDA in patients with IBD, ICER comparing FDI and FCM was €6590/QALY. Patients receiving FDI required on average 11.1 iron infusions per patient over ten years, compared with 14.2 infusions for those treated with FCM, 3.1 fewer infusions per patient. This implies that in an infusion service with fixed capacity, for every 100 patients treated with FDI, about 78 patients with comparable iron requirements could be treated using FCM.

However, because of the low cost of IV iron administration in Slovenia, this reduction translated into only €95 in savings. In contrast, iron procurement costs were substantially higher for FDI, resulting in an incremental cost of €1166 over ten years, which more than offset the modest savings in IV iron administration costs and additional costs (€176) associated with hypophosphatemia in the FCM arm.

**Figure 1:**
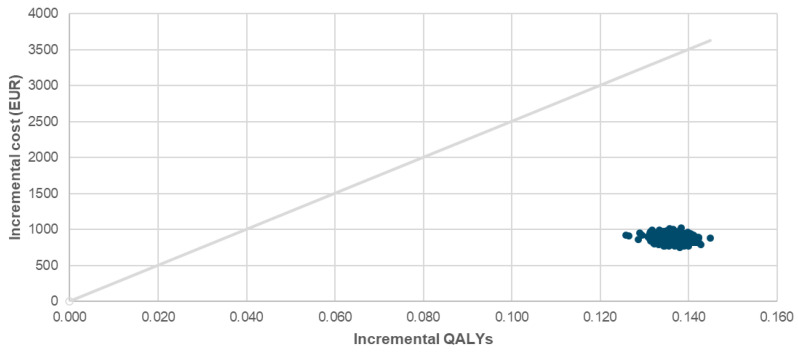
Probabilistic sensitivity analysis (PSA) scatter plot showing cost-effectiveness results when comparing FDI and FCM. QALY, quality adjusted life year; FCM, ferric carboxymaltose; FDI, ferric derisomaltose.

**Figure 2:**
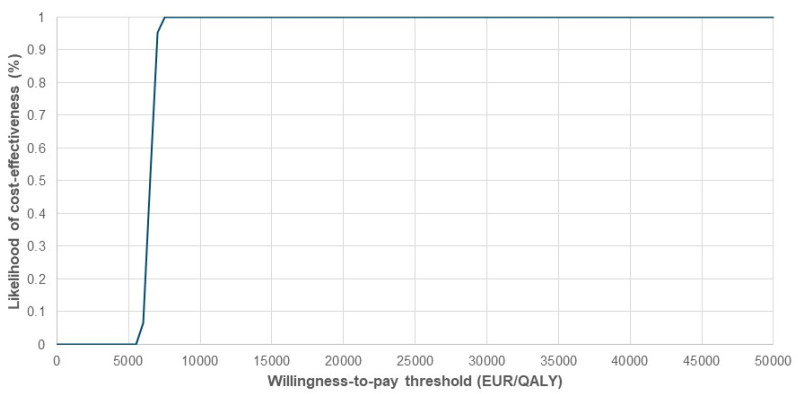
Cost-effectiveness acceptability curve of FDI compared to FCM. QALY, quality-adjusted life years; FCM, ferric carboxymaltose; FDI, ferric derisomaltose.

**Figure 3:**
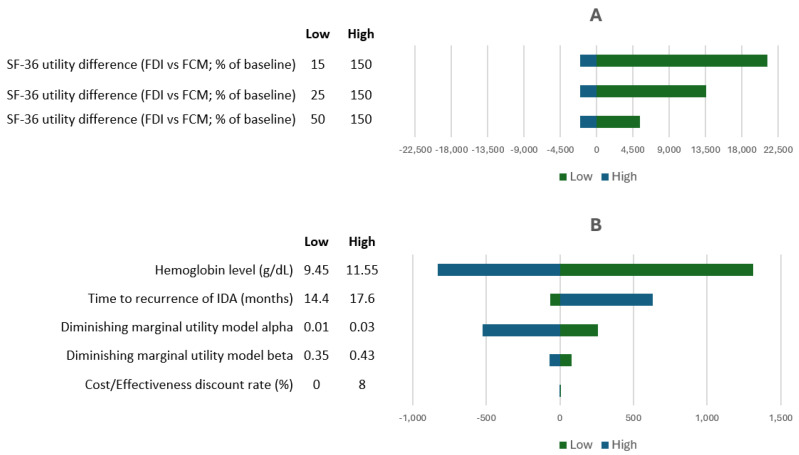
Summary of one-way sensitivity analysis in terms of change from baseline ICER (€/QALY). (**A**) Impact of SF-36 utility difference; (**B**) impact of other model parameters. ICER—Incremental cost-effectiveness ratio; QALY—quality-adjusted life year; IDA—iron deficiency anemia FCM, ferric carboxymaltose; FDI, ferric derisomaltose.

Figure 1 presents the cost-effectiveness plane comparing FDI and FCM. The PSA demonstrated a mean incremental QALY gain of 0.136, the same as in the deterministic base case, and a mean incremental cost of €875, closely matching the deterministic base case estimate of €895. This resulted in a mean ICER of €6198/QALY, compared to €6590/QALY in the deterministic base case. As shown in Figure 1, all iterations in PSA fell within the northeast quadrant of the cost-effectiveness plane but under WTP threshold of €25,000/QALY, confirming consistent cost-effectiveness. The cost-effectiveness acceptability curve (Figure 2) showed a 100% probability of cost-effectiveness at €8000/QALY. When FCM was modeled at zero acquisition cost, the ICER increased to €12,669/QALY (Table 2), yet remained below the Slovenian WTP threshold of €25,000/QALY, indicating the surprising robustness of the findings across cost settings. A sensitivity analysis using adjusted Danish IV iron administration costs (Table 2) further supported these findings, yielding an ICER of €4358/QALY. Results of a one-way sensitivity analysis are presented in Figure 3. When the clinical relevance of hypophosphatemia was minimized by applying a substantially reduced quality-of-life impact (15–50% of the baseline utility difference between FDI and FCM derived from SF-36 data), the resulting ICERs ranged from €11,938 to €27,719 per QALY. Under this scenario, the pharmacoeconomic advantage of FDI was attenuated, providing a lower-bound estimate of its cost-effectiveness.

### 3.2. Umbrella Review of Systematic Literature Reviews and Targeted Search of Consensus Statements

This review had two main objectives: (1) to identify systematic reviews reporting data on IV iron-induced hypophosphatemia and (2) to identify recommendations or consensus statements on its management. A comprehensive literature review was conducted to find potentially relevant articles from MEDLINE/PubMed and EMBASE databases (see Appendix B). A total of 580 records were identified (PubMed: 154, EMBASE: 426). After removing 176 duplicates, 404 records were screened by title and abstract. Of these, 344 were excluded as irrelevant, leaving 60 articles for full-text assessment, of which 12 met the inclusion criteria and were selected for data extraction. To ensure that no recently published or poorly indexed studies were missed, a brief, targeted supplementary search was performed after the primary PubMed and EMBASE search. During this search we identified two additional reviews [22,63] that were included in this umbrella review. Exclusion of two studies deserves a special note. One study [65] was excluded because it focused exclusively on trials directly comparing FCM with iron sucrose, while another study [66] examined only studies comparing oral versus IV iron during gastrointestinal bleeding; both were considered too narrowly scoped for the purposes of our analysis.

**Figure 4:**
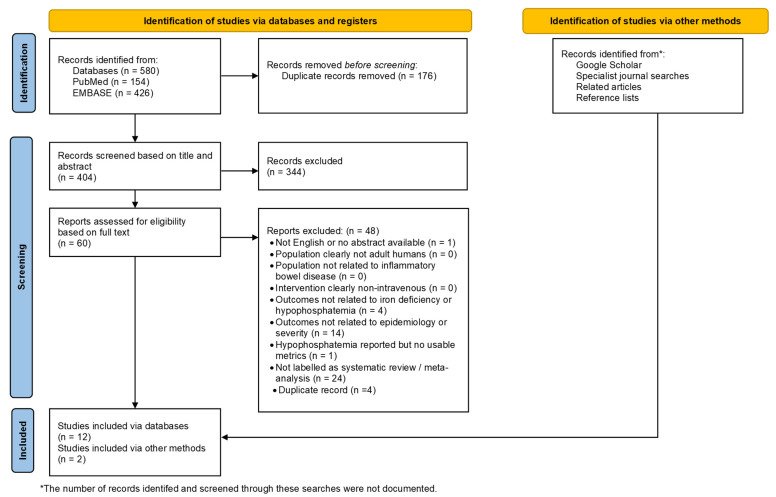
PRISMA flow chart of the umbrella review.

In total, 14 studies (12 from database searches and 2 from supplementary database sources) [10,11,19,24,31,67,68,69,70,71,72,73,74,75] constitute the evidence base of this umbrella review. The process of study identification, screening, and inclusion is illustrated in the PRISMA flow chart (Figure 4). This diagram provides a step-by-step summary of the number of records retrieved, screened, excluded, and finally included in the evidence base.

In addition to the umbrella review, a targeted literature search was also conducted to identify papers about the clinical management of hypophosphatemia associated with FCM. This search was intentionally restricted to consensus statements, clinical recommendations, and expert guidance documents, using predefined terms related to “FCM”, “hypophosphatemia”, “monitoring”, and “clinical management”. Primary studies and case reports were excluded to ensure a focus on clinical guidance. Eight recommendations and consensus papers were identified [22,29,32,76,77,78,79,80]. The selected studies were systematically organized based on whether they were systematic reviews or recommendations/consensus statements (Table 3). We report the results in the form of a narrative summary.

#### 3.2.1. Systematic Reviews

Early reviews primarily addressed the efficacy of IV iron therapy and either did not mention hypophosphatemia [81,82,83,84] or only mentioned it in passing [85]. Avni et al. [67] quantified IV iron use with increased risk of hypophosphatemia, while both Rognoni et al. [68] and Aksan et al. [69] offered only scanty evidence on incidence of hypophosphatemia in patients treated with IV iron.

Zoller et al. [24] provided one of the earliest overviews of IV-iron-induced hypophosphatemia as a clinically relevant and potentially long-term complication. Across 17 analyzed studies, hypophosphatemia occurred in 58% (95% confidence interval 42–74%; based on three studies) of FCM-treated patients with normal kidney function, higher than with other IV iron formulations. The authors also summarized 29 documented clinical cases linking FCM, iron polymaltose, and saccharated iron oxide to severe complications, such as osteomalacia and fractures.

Glaspy et al. [10] identified 40 studies (19 RCTs, 10 retrospective or observational studies, and 11 case reports) that investigated hypophosphatemia associated with IV iron therapies for IDA. Reported incidence varied widely across formulations, ranging from 0 to 92% with FCM, 0–40% with iron sucrose, 0.4% with ferumoxytol, and none with LMWID. Case reports described severe and chronic hypophosphatemia leading to fatigue, bone pain, osteomalacia, and fractures, particularly after repeated FCM dosing.

Bellos et al. [11] conducted a Bayesian network meta-analysis of eight randomized controlled trials (n = 5989) to compare the risk of hypophosphatemia among IV iron formulations used for treating IDA. The analysis showed that FCM was associated with higher incidence of hypophosphatemia than FDI (RR 7.9), iron sucrose (RR 9.4), LMWID (RR 6.6), and ferumoxytol (RR 24.0). FCM ranked worst overall, with a surface under the cumulative ranking curve (SUCRA) of 99.1%, indicating the highest likelihood of causing hypophosphatemia. Median incidence across studies was 45% for FCM versus <5% for other agents. Severe (serum phosphate < 0.42 mmol/L or 1.3 mg/dL) and persistent hypophosphatemia (serum phosphate < 0.65 mmol/L or 2 mg/dL ≥ 2 weeks post-treatment) occurred mainly after FCM administration.

Rosano et al. [70] performed a post hoc pooled analysis of 45 interventional trials (n = 15,080; 8245 received FCM) to evaluate the frequency, duration, and clinical relevance of hypophosphatemia following FCM treatment. This pooled analysis confirmed that FCM is associated with an increased rate of serum phosphate reduction. Among 6879 patients receiving FCM therapy, 41.4% experienced phosphate levels < 0.81 mmol/L (2.5 mg/dL) and 0.7% dropped below 0.32 mmol/L (1 mg/dL). Serum phosphate declined most markedly at 2 weeks post-infusion (mean −0.4 mmol/L or −1.24 mg/dL) and generally normalized by week 8–12. Hypophosphatemia was more frequent in gastroenterology and women’s health populations and among those receiving multiple or higher cumulative FCM doses (>1000 mg).

Schaefer et al. [71] conducted a systematic review and meta-analysis of 42 prospective clinical trials to compare the incidence, severity, and duration of hypophosphatemia following treatment with FCM versus FDI. The pooled analysis showed higher risk of hypophosphatemia with FCM (47%, 95% CI 36–58%) compared with FDI (4%, 95% CI 2–5%), and a greater mean serum phosphate reduction (−0.40 vs. −0.06 mmol/L). Hypophosphatemia persisted for up to three months in 45% of FCM-treated patients. Meta-regression identified low baseline ferritin, low transferrin saturation, and normal kidney function as predictors of hypophosphatemia.

In 2021, Glaspy et al. [31] built upon their earlier review [10], identifying 20 RCTs published between 2008 and 2020 that reported serum phosphate outcomes, 19 of which involved FCM. Across these studies, the incidence of hypophosphatemia ranged from 40% to 70%, with moderate and severe cases (phosphate < 2.0 mg/dL or <0.65 mmol/L), occurring in roughly up to half of FCM-treated patients, and severe cases (<1 mg/dL or 0.32 mmol/L) up to roughly 10%. The review indicated that hypophosphatemia often persists for weeks to months, occasionally leading to osteomalacia and fractures, especially after repeated dosing. The authors also summarized 15 newly reported clinical cases of symptomatic iron-induced hypophosphatemia published since the 2017 review by Zoller et al. [24], most involving patients with IDA repeatedly treated with FCM, although a few received iron polymaltose or iron sucrose. About half of the cases involved long-term FCM exposure (up to 4 years), with several developing osteomalacia and multiple fractures, especially among patients with Crohn’s disease.

The systematic review by Vilaca et al. [72] examined 28 case reports (30 cases total) of osteomalacia linked to repeated IV iron infusions. Most patients (n = 18) received FCM, with others given SFO (n = 8) or iron polymaltose (n = 3); in one patient the iron therapy was not reported. The majority had underlying gastrointestinal disorders, often requiring prolonged iron therapy. Across cases, persistent hypophosphatemia (median 0.36 mmol/L) and elevated FGF-23 were consistently observed, accompanied by high alkaline phosphatase, bone pain, and fractures or pseudofractures. Imaging typically revealed focal isotope uptake consistent with osteomalacia. Symptoms improved primarily after discontinuing or switching iron formulations, whereas vitamin D or phosphate supplementation offered limited benefit.

Rosano et al. [73] conducted another pooled analysis of 41 clinical trials including 7931 adults treated with FCM, this time to evaluate the risk of hypophosphatemia in heart failure (HF) compared to other conditions. Among the cohort, 14% had HF, and the incidence of moderate or severe hypophosphatemia (serum phosphate < 0.65 mmol/L or 2.0 mg/dL) was 8.1% in HF patients versus much higher rates in other groups, such as 30–55% in women’s health, gastrointestinal, and neurology populations. Only one HF patient (<0.1%) developed severe hypophosphatemia (<0.32 mmol/L or 1.0 mg/dL), compared with 4.8% and 4.0% of the subjects in the neurology and gastrointestinal groups, respectively. Serum phosphate levels typically decreased at weeks 2–4 and returned to baseline by week 8, mirroring findings in other subgroups. Preserved kidney function was identified as the strongest predictor of hypophosphatemia (odds ratio 12.2 for eGFR > 60 vs. <30 mL/min/1.73 m^2^). The study concluded that FCM-associated hypophosphatemia in HF is infrequent, transient, and rarely symptomatic, likely due to coexisting renal impairment limiting phosphate excretion, though monitoring remains advisable in cases of repeated dosing or high cumulative exposure. A recent study [86] though reported a hypophosphatemia incidence of 51% following FCM administration in HF patients. This discrepancy may reflect differences in assessment timing, as patients in Rosano et al. [73] were not systematically monitored and many were evaluated after the 2-week nadir period, when phosphate levels may have already begun to recover.

Malireddi et al. [74] performed a systematic review of 14 studies (five randomized controlled trials and nine observational studies, n = 2493) to assess the safety and efficacy of FCM for treating IDA in IBD patients. The incidence of hypophosphatemia after FCM infusion ranged from 21% to nearly 73% in various trials, typically peaking within two weeks post-infusion, with some patients remaining hypophosphatemic for over a month.

Magagnoli et al. [19] conducted a comprehensive systematic review of clinical trials, observational studies, case reports, and FDA adverse event data to evaluate the incidence, mechanisms, and clinical outcomes of FCM-associated hypophosphatemia. The review found that hypophosphatemia is a clinically significant adverse drug reaction linked to FCM and occurring at higher rates than with other IV iron formulations: 50–92% with FCM compared to 2–8% with other agents. Across 42 clinical trials and real-world datasets, FCM induced a greater and longer-lasting drop in phosphate levels than FDI or ferumoxytol, with up to 45% of affected patients remaining hypophosphatemic for three months or longer. Analysis of FDA’s Adverse Event Reporting System (FAERS) database (2014–2023) identified 1270 reports of FCM-associated hypophosphatemia, with an increasing trend over time, though the authors estimated that <1% of actual cases had been reported.

Galigutta et al. [75] undertook a large pharmacovigilance analysis and systematic review to evaluate serious adverse events associated with FCM. Using data from FAERS and VigiBase between 2003 and 2024, the study found strong disproportionality signal linking FCM to hypophosphatemia (proportional reporting ratio [PRR] of 520.7 in FAERS and 245.1 in VigiBase). The literature review of 11 case reports confirmed these findings, describing cases of prolonged, severe hypophosphatemia, osteomalacia, and fractures, as well as occasional life-threatening hypersensitivity reactions.

#### 3.2.2. Recommendations and Consensus Statements

Kassianides and Bhandari [22] examined IV-iron-induced hypophosphatemia and proposed a practical clinical algorithm to guide IV iron selection and hypophosphatemia management. They concluded that although modern IV iron formulations are generally safe and effective, FCM is associated with a distinct and higher risk of hypophosphatemia, warranting careful patient selection, biochemical monitoring, and consideration of alternative preparations in at-risk patients.

Boots and Quax [29] proposed evidence-based recommendations for the evaluation and management of hypophosphatemia in patients receiving IV iron. Their flowchart emphasized that, when FCM is used, the SmPC advises consideration of serum phosphate monitoring before administration and again after 14 days; however, because the nadir following a single FCM dose typically occurs at around 7 days, earlier monitoring is appropriate. The authors note that even patients deemed low risk may develop severe hypophosphatemia with FCM and suggest careful selection of the iron formulation. If iron deficiency cannot be corrected with a single 1000 mg dose and multiple infusions are expected, FCM may be unsuitable as repeated dosing can deepen and prolong hypophosphatemia and potentially increase the risk of osteomalacia. For patients at elevated risk, clinicians should consider formulations with lower reported hypophosphatemia risk than FCM, like iron sucrose, ferumoxytol, and FDI. Boots and Quax [29] further emphasized that prevention is preferable to treatment and that phosphate supplementation, oral or IV, should be reserved for clinically urgent situations due to its limited effectiveness and its potential adverse effects.

Schaefer et al. [32] provided diagnostic and management recommendations, emphasizing serum phosphate monitoring in patients requiring repeated or high-dose FCM or those with unexplained fatigue, bone pain, or muscular weakness. In affected patients, they recommend assessing calcium, PTH, alkaline phosphatase, and vitamin D metabolites, along with imaging studies to detect osteomalacia (e.g., looser zones on X-ray or scintigraphy). For prevention, clinicians are advised to delay additional FCM doses until recovery, consider switching to iron formulations with lower hypophosphatemia risk, and manage complications with active vitamin D analogs to mitigate secondary hyperparathyroidism.

**Figure 5:**
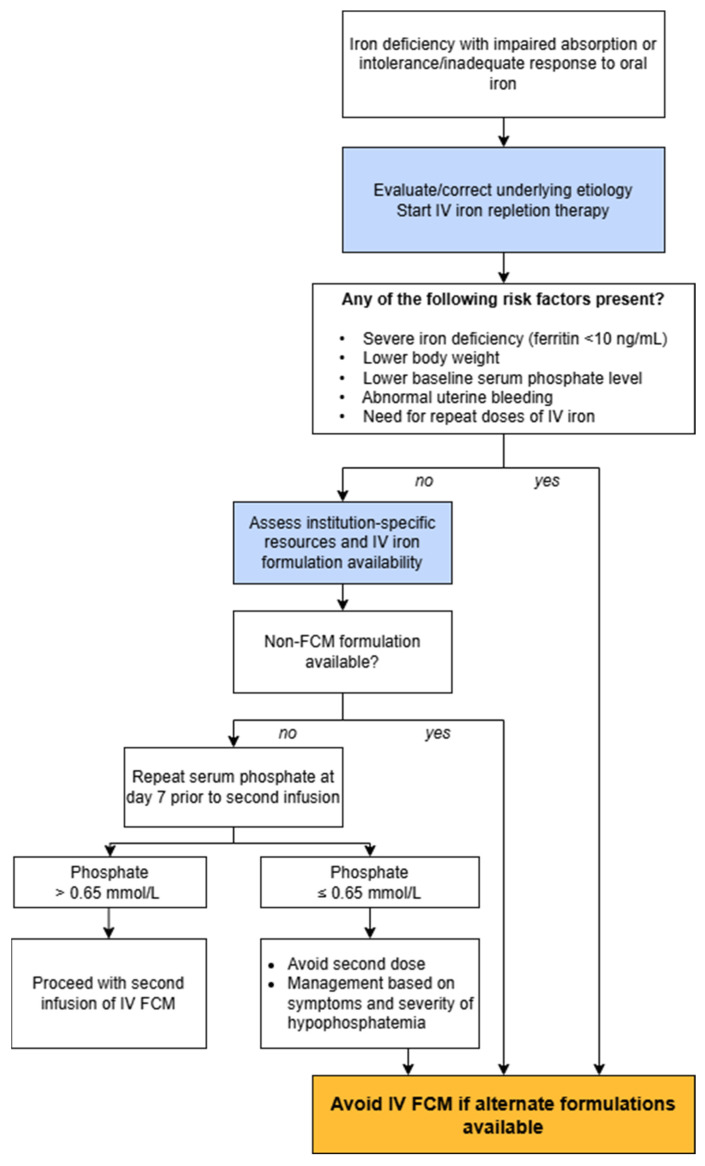
Algorithm for management and prevention of hypophosphatemia. The threshold value of 0.65 mmol/L corresponds to 2.0 mg/dL. Adapted from Martens and Wolf [76]. FCM—ferric carboxymaltose; IV—intravenous.

Martens and Wolf [76] provided an evidence-based mini-review on the incidence, mechanism, and clinical management of IV-iron-induced hypophosphatemia, focusing on FCM as the principal cause. They outlined that FCM uniquely induced hypophosphatemia occurring in up to 75% of recipients through an acute three- to sixfold increase in FGF-23, which causes increased renal phosphate wasting, reduced 1,25-dihydroxyvitamin D synthesis, and secondary hyperparathyroidism. The article identified normal kidney function, severe iron deficiency, need for repeated doses of IV iron, low body weight, low baseline phosphate, and abnormal uterine bleeding as risk factors. Clinical symptoms can include fatigue, myalgia, bone pain, weakness, or “brain fog,” and chronic cases may progress to osteomalacia or fractures with repeated dosing of FCM. The authors provided management and prevention recommendations summarized in the algorithm (Figure 5). They advised avoiding FCM when alternative formulations such as ferumoxytol or FDI are available, particularly in patients requiring repeated infusions. When FCM use is unavoidable, they recommend serum phosphate testing at week 1 before administering a second dose, withholding treatment if phosphate levels are ≤0.65 mmol/L (or ≤2.0 mg/dL), and evaluating for hypophosphatemia in any patient presenting with fatigue, myalgia, or bone pain after infusion. The authors indicate that phosphate and vitamin D supplementation are often limited in effectiveness in the setting of ongoing FGF-23 excess, underscoring prevention and early detection as the most effective strategy.

Van Doren et al. [77] published an expert consensus guideline on the safe use, administration, and monitoring of IV iron formulations, focusing on the recognition and management of infusion reactions and treatment-emergent hypophosphatemia. With respect to hypophosphatemia, the consensus emphasizes that FCM poses the highest risk, with reported incidence rates between 47% and 75%, while other formulations, such as ferumoxytol, FDI, and LMWID are associated with a substantially lower risk of hypophosphatemia and rarely with clinically significant or persistent phosphate depletion. The authors recommend avoiding FCM in patients requiring repeated infusions or with chronic blood loss or malabsorptive disorders, as prolonged use can lead to osteomalacia and fractures. Routine phosphate monitoring is primarily recommended for FCM users, particularly prior to repeat dosing within three months, whereas other formulations warrant symptom-driven testing. Importantly, phosphate or vitamin D supplementation is largely ineffective, and management should focus on cessation of FCM and prevention of secondary hyperparathyroidism.

Fraser et al. [78] presented a nursing consensus paper that established 16 evidence-based recommendations on the practical management of infusion reactions and hypophosphatemia associated with IV iron therapy. The paper specifically provided recommendations on monitoring and prevention of hypophosphatemia. Nurses are advised to watch for fatigue [87], weakness, and bone pain that may develop days to weeks after infusion and to measure serum phosphate before and after treatment in patients at risk or receiving multiple doses of FCM.

Rosano et al. [79] presented an international multidisciplinary consensus on the risk assessment and management of hypophosphatemia associated with FCM. The expert panel, comprising specialists from cardiology, nephrology, hematology, endocrinology, and molecular biology, concluded that in many clinical settings, particularly cardiology populations, FCM-associated hypophosphatemia is often transient, asymptomatic, and self-limiting, typically resolving within eight weeks without specific intervention. However, the authors also acknowledged that the incidence of hypophosphatemia is higher with FCM than with other IV iron formulations and that recurrent or prolonged hypophosphatemia, especially with repeated dosing, may result in clinically relevant sequelae, including osteomalacia.

Compared with prior recommendations, including those of Martens and Wolf [76] and Van Doren et al. [77], and Rosano et al. [79], adopted a more selective approach to phosphate monitoring. Whereas Martens and Wolf [76] advocated routine serum phosphate testing approximately one week after FCM administration and avoidance of repeat dosing when phosphate levels are ≤0.65 mmol/L (or ≤2.0 mg/dL), Rosano et al. [79] recommended targeted testing primarily in high-risk or symptomatic individuals, and in patients requiring repeated or higher-dose infusions. In contrast to Van Doren et al. [77], who advised avoiding FCM in patients needing recurrent infusions, this consensus did not discourage its use outright but rather emphasized individualized risk assessment and the low incidence of serious sequelae such as osteomalacia. Overall, Rosano et al. [79] frame FCM as an effective therapy in many clinical settings and propose a less prescriptive framework for managing FCM-associated hypophosphatemia.

The Scandinavian expert consensus by Detlie et al. [80] recognized hypophosphatemia as a safety concern associated with IV iron therapy in patients with IDA and IBD. The panel highlighted the higher risk of hypophosphatemia with FCM compared with other formulations and supported consideration of FDI as an alternative, particularly when repeated dosing is anticipated.

## 4. Discussion

In this study, we evaluated the cost effectiveness of FDI versus FCM for treating IDA in patients with IBD within the Slovenian healthcare system. The pharmacoeconomic model showed that FDI remains cost effective and below the national ICER threshold even under a highly unfavorable pricing scenario in which FCM is priced at 42% of FDI. As described in the Methods section (Section 2), several clinically relevant consequences of hypophosphatemia, such as bone and muscle pain, or osteomalacia with (pseudo)fractures, were not incorporated into the model; their exclusion likely leads to an underestimation of the full clinical and economic impact of hypophosphatemia and therefore renders the analysis conservative.

Beyond the base modeling results, this work integrates pharmacoeconomic modeling with an umbrella review of systematic reviews and expert consensus statements. Together, these elements provide an up-to-date perspective that reflects Slovenia’s current cost environment and evolving clinical guidance on IV-iron-related hypophosphatemia. Slovenia may serve as an informative case example for CEE countries, given its advanced and transparent pharmaceutical reimbursement framework and broad market access to innovative therapies [55]. A similar evaluative approach can also be applied beyond pharmaceuticals, including to medical devices and emerging diagnostic technologies [88,89,90,91,92,93,94].

### 4.1. Economic Interpretation

The pharmacoeconomic modeling demonstrated that FDI remains cost effective compared with FCM despite Slovenia’s unusually low infusion costs and the recent substantial price reduction in FCM. The base-case ICER of €6590/QALY was below the national WTP threshold of €25,000/QALY, indicating that FDI provides value for money even under conservative modeling assumptions. When FCM was modeled at zero acquisition cost, the ICER increased to €12,669/QALY yet remained cost effective, underscoring the robustness of results across price scenarios. However, it should be acknowledged that the markedly lower price of FCM compared with FDI makes it highly appealing from a short-term hospital budget perspective, where tender decisions are typically driven by unit acquisition price rather than formal cost-effectiveness criteria [95]. In this context, procurement processes emphasize immediate budgetary impact over long-term clinical or economic value, such that lower-cost options are favored even when they entail higher downstream risks or monitoring requirements. This dynamic highlights the value of structured health technology assessment (HTA) approaches within hospital decision-making to ensure that considerations of safety, resource use, and patient outcomes are not overshadowed by unit-price pressures [95].

Our findings contrast with previous analyses in Western Europe [48,49,50,96], where higher infusion costs combined with more comparable pricing of FDI and FCM amplified the economic advantage of FDI. In the Slovenian setting, the substantial cost differential between the two drugs outweighed the minor savings associated with fewer infusions required for FDI. Nevertheless, because the model explicitly incorporated the health-related quality-of-life impact of hypophosphatemia, FDI remained the cost-effective option. An important area for future refinement is the treatment of hypophosphatemia within the model. Hypophosphatemia varies widely in severity and duration, and accounting for the transient nature of the condition and its spectrum of severity would improve the accuracy of the model.

### 4.2. Clinical Evidence and Recommendations

The umbrella review of systematic literature reviews and consensus statements confirmed marked differences in the incidence of hypophosphatemia between IV iron formulations. According to studies, FCM exhibited the highest reported incidence (up to 92%), whereas other preparations, such as ferumoxytol, LMWID, and FDI generally demonstrated rates below 10%. Severe or persistent hypophosphatemia following FCM has been associated with clinically relevant sequelae, including osteomalacia, fractures, or chronic fatigue, largely attributable to FGF-23-mediated renal phosphate wasting and secondary hyperparathyroidism. While many cases are transient and reversible, prolonged exposure or repeated dosing has been associated with sustained metabolic disturbances. With the exception of the more permissive consensus by Rosano et al. [79], expert recommendations have been broadly consistent over recent years [32,76,77,78,80]. These recommendations generally support routine post-FCM serum phosphate testing, particularly prior to repeat dosing, and the avoidance of additional FCM administration when phosphate levels are ≤0.65 mmol/L (≤2.0 mg/dL), as well as discouraging the use of FCM in patients requiring frequent or long-term infusions.

### 4.3. Potential Clinical Consideration

A potential clinical consideration is whether patients should undergo baseline serum phosphate testing before IV iron administration. A subset of individuals with IBD, particularly those with active disease or ongoing inflammation, may already have hypophosphatemia prior to infusion and identifying such patients in advance may help prevent exacerbation or prolongation of phosphate depletion following IV iron therapy.

### 4.4. Comparison with Other Umbrella Reviews

Netzer et al. [97] provided a comprehensive synthesis of evidence on phosphate testing and supplementation in adults outside intensive care settings across a range of clinical conditions, such as X-linked hypophosphatemia, tumor-induced osteomalacia, and post-kidney-transplantation states. Among the 33 publications reviewed (11 guidelines, 19 reviews, 3 consensus statements), only three specifically addressed hypophosphatemia associated with FCM [29,32,72]. All three of these publications were included in the present review.

### 4.5. Limitations

Several limitations should be acknowledged. Pharmacoeconomic analysis was conducted from the payer perspective and did not include indirect costs, such as productivity loss, to maintain a conservative approach. Similarly, fracture-related outcomes were excluded from the model, likely underestimating the long-term burden of severe hypophosphatemia. Evidence on the long-term clinical consequences of phosphate depletion remains limited, highlighting the need for further prospective and retrospective studies, to refine future cost-effectiveness estimates.

Certain model inputs and assumptions relied exclusively on findings from the PHOSPHARE-IBD study, which may introduce a potential source of bias. In particular, the assumption of equivalent hematological responses and comparable hemoglobin increases between FDI and FCM was derived directly from PHOSPHARE-IBD. The umbrella review also has methodological limitations, as it relied on a narrative synthesis rather than a quantitative meta-analysis. This approach was deliberately chosen to accommodate the umbrella review format, which encompassed both systematic reviews and expert recommendations or consensus statements. This allowed the integration of diverse sources of evidence while maintaining transparency and breadth. It is important to note that all limitations of the studies included in the umbrella review also apply to the present analysis. Finally, patient and public involvement in disease assessment has until recently received limited attention [98]. Systematic inclusion of patient perspectives could further strengthen our study, improve alignment with patient needs, and enhance overall societal relevance.

## 5. Conclusions

In summary, this modeling study showed that FDI remains a cost-effective alternative to FCM for treating IDA in patients with IBD within the Slovenian healthcare setting, primarily due to its more favorable hypophosphatemia profile within the model. The finding suggests that the risk of hypophosphatemia should be considered when selecting IV iron therapy in routine IBD care.

Our analysis emphasizes that hypophosphatemia remains an underrecognized yet clinically meaningful adverse effect of IV iron therapy. Its symptoms, such as fatigue, muscle weakness, and dyspnea, may closely resemble those of untreated anemia, potentially leading to delayed recognition and management. Particular caution is warranted in patients with osteoporosis, vitamin D deficiency, malabsorptive conditions, or in those requiring repeated or high-dose FCM infusions, as these populations are at increased risk of clinically relevant phosphate depletion. In cases of severe or persistent hypophosphatemia, referral to specialists in bone metabolism may be appropriate to manage complications such as osteomalacia.

## Figures and Tables

**Table 1:** Summary of model parameters.

Cohort Characteristics			
	Mean	SD	Reference
Age (years)	42.10	14.40	Zoller et al. [12]
Hemoglobin (g/dL)	10.50	1.40	Zoller et al. [12]
Body mass (kg)	80.20	15.90	Zoller et al. [12]
	**Median**	**95% CI**	**Reference**
Time to recurrence of IDA (months)	16	7–24	Kulnigg et al. [59]
**Quality of life**			**Reference**
Diminishing marginal utility model alpha	0.0161		Hu et al. [60]
Diminishing marginal utility model beta	0.3922		Hu et al. [60]

**Table 2:** Summary of reported outcomes for various scenarios in the study. QALY, quality-adjusted life year; FCM, ferric carboxymaltose; FDI, ferric derisomaltose; ICER—Incremental cost-effectiveness ratio; IV—intravenous.

	Quality-Adjusted Life Expectancy (QALYs)			Costs (€)		
Scenario	FDI	FCM	Difference	FDI	FCM	Difference
Base case	4.862	4.726	0.136	2329	1434	895
ICER (€/QALY)	6590					
FCM at zero cost	4.862	4.726	0.136	2329	608	1721
ICER (€/QALY)	12,669					
Adjusted Danish IV iron administration costs	4.862	4.726	0.136	3402	2810	592
ICER (€/QALY)	4358					

**Table 3:** Articles included in the umbrella review [10,11,19,24,31,67,68,69,70,71,72,73,74,75] and consensus statements identified through targeted search [22,29,32,76,77,78,79,80]. HPP—hypophosphatemia; FCM—ferric carboxymaltose; FDI—ferric derisomaltose; SFO—saccharated ferric oxide; IPM—iron polymaltose; HF—heart failure; IBD—inflammatory bowel disease; PRR—proportional reporting ratio; FAERS—FDA Adverse Event Reporting System; CI—confidence interval; LMWID—low-molecular-weight iron dextran; RR—relative risk.

Reference	Year of Publication	Number of Included Studies	Major Conclusions
Systematic reviews			
Avni et al. [67]	2015	103	IV iron use “was associated with an increased risk of electrolyte disorder (most trials reported on the occurrence of HPP) (RR, 2.45; 95% CI, 1.84–3.26)” [67]
Rognoni et al. [68]	2016	21	Out of 21 studies, HPP reported only in one, with an incidence from 3.7 to 5.5% in patients receiving FCM
Aksan et al. [69]	2017	5	Of 543 patients on FCM, 1.7% experienced HPP
Zoller et al. [24]	2017	17	HPP occurred in 58% (95% CI 42–74%; based on three studies) of FCM-treated patients with normal kidney function
Glaspy et al. [10]	2020	40	Incidence of HPP ranging from 0 to 92% for FCM, 0–40% for iron sucrose, 0.4% for ferumoxytol, and none for LMWID
Bellos et al. [11]	2020	8	Median incidence of HPP across studies 45% for FCM versus <5% for other agents
Rosano et al. [70]	2020	45	Among patients receiving FCM therapy, 41.4% with HPP and 0.7% with severe HPP
Schaefer et al. [71]	2021	42	Higher risk of HPP for FCM (47%, 95% CI 36–58%) compared to FDI (4%, 95% CI 2–5%)
Glaspy et al. [31]	2021	20	Incidence of HPP ranged from 40% to 70% for patients receiving FCM
Vilaca et al. [72]	2022	30	28 case reports (30 cases total) of osteomalacia linked to repeated intravenous iron infusions, with patients receiving FCM (n = 18), SFO (n = 8) or IPM (n = 3), and one was not reported; in case series, the lowest phosphate levels were between 0.16 and 0.77 mmol/L, and one patient had mild, 20 had moderate, and 8 had severe HPP (one was not reported); the cut-offs of 0.8, 0.6, and 0.3 mmol/L to categorize mild, moderate, or severe HPP were used
Rosano et al. [73]	2023	41	Only one HF patient (<0.1%) developed severe HPP (<0.32 mmol/L or 1.0 mg/dL), compared to 4.8% and 4.0% of the subjects in the neurology and gastrointestinal groups, respectively; the prevalence of moderate or severe HPP among the women’s health, other, gastrointestinal, and neurology subgroups was 30.1%, 40.6%, 51.0%, and 55.6%, respectively
Malireddi et al. [74]	2024	14	Incidence of HPP after FCM infusion ranged from 21% to nearly 73% in IBD patients
Magagnoli et al. [19]	2025		HPP of 50–92% for FCM compared to 2–8% for other agents
Galigutta et al. [75]	2025		Strong disproportionality signal linking FCM to HPP (PRR of 520.7 in FAERS and 245.1 in VigiBase)
**Recommendations and consensus statements**			
Kassianides and Bhandari [22]	2021		Clinical algorithm for IV iron use and the management of hypophosphatemia (Figure 4 in [22]).
Boots and Quax [29]	2022		Flowchart for safe use of IV iron formulations (Figure 3 in [29]).
Schaefer et al. [32]	2022		Summary of biochemical manifestations of hypophosphatemia following FCM administration (Figure 5 in [32]).
Martens and Wolf [76]	2023		Algorithm for the selection and safe administration of IV FCM to minimize the risk of hypophosphatemia (Figure 2 in [76]).
Van Doren et al. [77]	2024		Consensus-based recommendations for the IV iron use and management of adverse reactions (Table 1 in [77]).
Fraser et al. [78]	2025		Validated tools for the assessment of fatigue in clinical practice (Table 2 in [78]).
Rosano et al. [79]	2025		Proposed approach for evaluating the risk of hypophosphatemia in patients treated with FCM (Figure 1 in [79]),
Detlie et al. [80]	2025		Overview of the stages of iron deficiency, with anemia as a consequence of severe iron depletion (Figure 3 in [80]).

## Data Availability

The raw data supporting the conclusions of this article will be made available by the authors on request.

## Abbreviations

The following abbreviations are used in this manuscript:

CEE	Central and Eastern Europe
CI	Confidence interval
CU	Cost utility
ECCO	European Crohn’s and Colitis Organization
FAERS	FDA Adverse Event Reporting System
FCM	Ferric carboxymaltose
FDI	Ferric derisomaltose
FGF-23	Fibroblast growth factor 23
HF	Heart failure
HTA	Health technology assessment
IBD	Inflammatory bowel disease
ICER	Incremental cost-effectiveness ratio
IDA	Iron-deficiency anemia
IV	Intravenous
LMWID	Low-molecular-weight iron dextran
MHRA	Medicines and Healthcare products Regulatory Agency
PRR	Proportional reporting ratio
PSA	Probabilistic Sensitivity Analysis
QALY	Quality-Adjusted Life Year
QoL	Quality of Life
RCT	Randomized controlled clinical trial
SE	Standard Error
SmPC	Summary of product characteristics
WTP	Willingness-To-Pay

## Appendix A. CU-IDA Model

The conceptual foundation of the CU-IDA Model has been described in detail elsewhere [47,48,49,50]. In this study, the patient-level micro-simulation model was adapted to evaluate CU of FDI versus FCM from the Slovenian national payer perspective. A microsimulation approach [50], a standard method also applied in other therapeutic domains [99], was selected over a cohort model due to the non-linear relationship between patient characteristics and iron requirements. Developed in accordance with best-practice standards, the model incorporated patient-level heterogeneity, first-order uncertainty, and parameter-level stochastic variation to robustly quantify uncertainty in outcomes [50].

Each of 2000 simulated patients with chronic IDA at baseline was assigned an age, body weight, and hemoglobin level, sampled independently from lognormal distributions. These parameters determined individual iron needs and the number of required infusions per cycle based on product characteristics for each IV iron formulation. Costs of iron administration and related quality-of-life (QoL) decrements were derived accordingly. The model reported quality-adjusted life years (QALYs) and costs, including iron procurement, administration, and phosphate monitoring. No indirect costs were considered as they are not recognized by the Slovenian payer. ICERs were derived to compare FDI and FCM; outcomes were averaged separately for FCM and FDI across all simulated patients.

As in previous models [47,48,49,50], baseline patient characteristics were derived from the PHOSPHARE-IBD RCT [12]. The cohort had a mean age (SD) of 42.1 (14.4) years, body weight of 80.2 (15.9) kg, and hemoglobin level of 10.5 (1.40) g/dL, all modeled assuming lognormal distribution. Patients received either FDI or FCM on days 1 and 35, following identical dosing protocols determined by body weight and hemoglobin levels. The median time to iron retreatment was set at 16 months (95% CI: 7–24 months) based on pooled data from IBD-associated IDA studies [59].

Baseline disease parameters were also derived from the PHOSPHARE-IBD RCT [12]. By day 14, the proportion of patients experiencing any hypophosphatemia (<2 mg/dL) was 2.2% in the FDI arm and 45.8% in the FCM arm, assuming uniform distribution. Severe hypophosphatemia (≤1 mg/dL) occurred by day 14 in 0.0% of patients receiving FDI and 2.0% of those treated with FCM, again assuming uniform distribution. To maintain a conservative approach, the model assumed that patients with hypophosphatemia did not develop hypophosphatemic osteomalacia or fractures. Baseline utility values derived from SFv36 data [48] are shown in Figure A1. Consistent with the PHOSPHARE-IBD RCT findings [12], the proportion of patients achieving a hematological response, and the rate at which this response occurred, was modeled identically for both arms. As in the previous models [47,48,49,50], diminishing marginal utility model [60] was employed to calculate the infusion-related process disutility for each iron infusion.

## Appendix B. Literature Review Methodology

### Appendix B.1. Objective and Research Question

The objective of this umbrella review was to assess, among adults with IBD treated for IDA using IV iron, the incidence and severity of hypophosphatemia and how these risks vary across different IV iron formulations. Specifically, the study aimed to: (a) identify and synthesize systematic reviews (with or without meta-analyses) reporting hypophosphatemia following parenteral iron therapy in IBD; (b) compare the frequency and severity of hypophosphatemia across IV iron formulations; and (c) summarize the duration and time course of phosphate changes, associated biochemical correlates, and any reported skeletal outcomes. The review question was structured according to the PICOS framework (Table A1) [91]. We focused on reviews involving adult human patients with IBD treated for IDA (Population) with IV iron formulations (Intervention), compared against other IV iron formulations (Comparator). Outcomes of interest included incidence of hypophosphatemia, duration/time to nadir, recurrent episodes; biochemical markers; clinically significant sequelae (Outcomes). Eligible study designs systematic reviews with or without meta-analysis, while primary studies, reviews, animal studies, ex vivo work, and non-peer-reviewed abstracts were excluded (Study design).

**Table A1:** PICOS criteria to define the research questions.

Population (P)	Adults (≥18 y) with IBD (Crohn’s disease or ulcerative colitis) treated for IDA (inpatient or outpatient). Mixed-population SLRs are eligible only if IBD-specific data or clearly separable gastrointestinal/IBD subgroup results are reported.
Intervention (I)	IV iron formulations (ferric carboxymaltose, ferric derisomaltose/iron isomaltoside, iron sucrose, ferumoxytol, low-molecular-weight iron dextran)
Comparator (C)	Other IV iron formulations; or pooled incidence without direct comparator (acceptable for adverse-event frequency).
Outcomes (O)	Incidence of hypophosphatemia (overall and by severity grade); duration/time to nadir, recurrent episodes; biochemical markers; clinically significant sequelae (bone pain, fractures, osteomalacia).
Study design (S)	Systematic reviews (with explicit methods: predefined question, systematic search, eligibility criteria, study selection) with or without meta-analysis. Primary studies, scoping/non-systematic/narrative reviews excluded.

### Appendix B.2. Search Strategy

The literature review was conducted and reported in compliance with the PRISMA 2020 Statement [64], a guide for standard reporting of systematic reviews (including umbrella reviews). The date of the search was 1 September 2025. Search strings in each database with the number of records are summarized in Table A2 and Table A3.

**Table A2:** Search strategy and number of hits in PubMed.

PUBMED			
	PICOS Elements	Search Terms	Number of Hits
#1	Population	((“Hypophosphatemia”[MeSH] OR “Phosphates/blood”[MeSH] OR hypophosphat*[tiab] OR hypophosphataem*[tiab] OR “hypo phosphate”[tiab] OR “low phosphate”[tiab] OR “low serum phosphate”[tiab] OR phosphat*[tiab]) OR (“Anemia, Iron-Deficiency”[MeSH] OR “iron deficiency”[tiab] OR “iron deficiency anemia”[tiab] OR IDA[tiab]))	646,798
#2	Intervention	(“intravenous iron”[tiab] OR “IV iron”[tiab] OR “parenteral iron”[tiab] OR “parenteral iron therapy”[tiab] OR “intravenous iron therapy”[tiab] OR “iron infusion”[tiab] OR “iron infusion therapy”[tiab] OR “iron replacement”[tiab] OR “iron replacement therapy”[tiab] OR “iron repletion”[tiab] OR “iron repletion therapy”[tiab] OR “iron infusion”[tiab] OR “iron infusion therapy”[tiab]) OR (“ferric carboxymaltose”[tiab] OR “carboxymaltose”[tiab] OR FCM[tiab]) OR (“ferric derisomaltose”[tiab] OR “derisomaltose”[tiab] OR FDI[tiab]) OR (“iron isomaltoside”[tiab] OR “isomaltoside”[tiab] OR “iron isomaltoside 1000”[tiab]) OR (“iron sucrose”[tiab] OR “iron sucrose complex”[tiab] OR “iron hydroxide sucrose”[tiab]) OR (ferumoxytol[tiab] OR FMX[tiab]) OR (“ferric gluconate”[tiab] OR “ferric gluconate complex”[tiab]) OR (“iron dextran”[tiab] OR “low molecular weight iron dextran”[tiab] OR “LMW iron dextran”[tiab]) OR (“iron polymaltose”[tiab] OR “polymaltose iron”[tiab] OR “iron polymaltose complex”[tiab]) OR (“saccharated ferric oxide”[tiab] OR “saccharated iron oxide”[tiab] OR “ferric hydroxide polymaltose”[tiab] OR “iron hydroxide polymaltose”[tiab]) OR (Ferinject[tiab] OR Injectafer[tiab] OR Monofer[tiab] OR Monoferric[tiab] OR Venofer[tiab] OR Feraheme[tiab] OR Ferrlecit[tiab] OR Cosmofer[tiab] OR Maltofer[tiab])	20,052
#3	Publication type	(meta-analysis[Publication Type] OR systematic review[Publication Type] OR systematic[sb])	463,360
#4	#1 AND #2 AND #3		154

**Table A3:** Search strategy and number of hits in EMBASE.

EMBASE			
	PICOS Elements	Search Terms	Number of Hits
#1	Population	((‘hypophosphataemia’/exp OR ‘phosphate’/exp OR hypophosphat*:ti,ab OR hypophosphataem*:ti,ab OR ‘hypo phosphate’:ti,ab OR ‘low phosphate’:ti,ab OR ‘low serum phosphate’:ti,ab OR phosphat*:ti,ab) OR ((‘iron deficiency anemia’/exp OR ‘iron deficiency anemia’/exp OR ‘anemia, iron deficiency’/exp) OR ‘iron deficiency’:ti,ab OR ‘iron-deficiency’:ti,ab OR ‘iron deficiency anemia’:ti,ab OR IDA:ti,ab))	821,580
#2	Intervention	((‘intravenous administration’/exp OR intravenous:ti,ab OR “IV iron”:ti,ab OR ‘parenteral iron’:ti,ab OR ‘parenteral iron therapy’:ti,ab OR ‘intravenous iron therapy’:ti,ab OR ‘iron infusion’:ti,ab OR ‘iron infusion therapy’:ti,ab OR ‘iron replacement’:ti,ab OR ‘iron replacement therapy’:ti,ab OR ‘iron repletion’:ti,ab OR ‘iron repletion therapy’:ti,ab) OR (‘ferric carboxymaltose’/exp OR ferric carboxymaltose:ti,ab OR carboxymaltose:ti,ab OR FCM:ti,ab) OR (‘ferric derisomaltose’/exp OR ferric derisomaltose:ti,ab OR derisomaltose:ti,ab OR FDI:ti,ab) OR (‘iron isomaltoside’/exp OR ‘iron isomaltoside 1000’:ti,ab OR isomaltoside:ti,ab) OR (‘iron sucrose’/exp OR ‘iron sucrose complex’:ti,ab OR ‘iron hydroxide sucrose’:ti,ab) OR (ferumoxytol/exp OR ferumoxytol:ti,ab OR FMX:ti,ab) OR (‘ferric gluconate’/exp OR ‘ferric gluconate complex’:ti,ab) OR (‘iron dextran’/exp OR ‘low molecular weight iron dextran’:ti,ab OR ‘LMW iron dextran’:ti,ab) OR (‘iron polymaltose’/exp OR ‘polymaltose iron’:ti,ab OR ‘iron polymaltose complex’:ti,ab) OR (‘saccharated ferric oxide’/exp OR ‘saccharated iron oxide’:ti,ab OR ‘ferric hydroxide polymaltose’:ti,ab OR ‘iron hydroxide polymaltose’:ti,ab) OR (Ferinject:ti,ab OR Injectafer:ti,ab OR Monofer:ti,ab OR Monoferric:ti,ab OR Venofer:ti,ab OR Feraheme:ti,ab OR Ferrlecit:ti,ab OR Cosmofer:ti,ab OR Maltofer:ti,ab))	886,095
#3	Filter applied	Study tpye: systematic literature review OR meta analysis	
#4	#1 AND #2 AND #3		426

### Appendix B.3. Screening Methods

#### Appendix B.3.1. Title and Abstract Screening

Screening was performed by one reviewer, with 20% of titles and abstracts independently checked by a second reviewer to ensure consistency. Discrepancies were resolved by discussion. Records were excluded if they met any of the following criteria (hierarchical), otherwise they progressed to the full-text screening stage:

1. Not English or no abstract available
2. Population clearly not adult humans
3. Population not related to inflammatory bowel disease
4. Intervention clearly non-intravenous
5. Outcomes not related to iron deficiency or hypophosphatemia
6. Outcomes not related to epidemiology or severity
7. Not labeled as systematic review/meta-analysis.

#### Appendix B.3.2. Full-Text Screening

Potentially eligible papers were retrieved in full and assessed by the same primary reviewer, with 20% double-checked. Exclusion reasons were recorded for the PRISMA diagram. Full texts were excluded if they met any of the following criteria, otherwise they were included:

1. Not English or no abstract available
2. Population clearly not adult humans
3. Population not related to inflammatory bowel disease
4. Intervention clearly non-intravenous
5. Outcomes not related to iron deficiency or hypophosphatemia
6. Outcomes not related to epidemiology or severity
7. Hypophosphatemia reported but no usable metrics
8. Not labeled as systematic review/meta-analysis
9. Duplicate.

#### Appendix B.3.3. Additional Sources

In addition to the database queries, we manually screened reference lists of key reviews, conference proceedings, and specialist journals. This hand-search yielded a small number of extra records that were not indexed in PubMed or EMBASE. These articles were imported into EndNote and processed according to the same de-duplication, screening, and data-extraction workflow described above.

#### Appendix B.3.4. Data Extraction

The primary reviewer extracted study characteristics, details on the investigated outcomes like incidence of hypophosphatemia (overall and by severity grade); duration/time to nadir, recurrent episodes; biochemical markers; clinically significant sequelae, etc., and key findings into a pre-piloted Excel sheet; in total, 20% of extractions were verified by the second reviewer. Conflicts were resolved by consensus. All steps and exclusions are summarized in a PRISMA-2020 flow diagram (Figure 4).

#### Appendix B.3.5. Screening Results

The database search retrieved 580 records in total (154 from PubMed and 426 from EMBASE). After automated and manual de-duplication in EndNote, 404 unique citations remained. Title and abstract screening removed 344 irrelevant or ineligible papers, leaving 60 articles for full-text assessment. Twelve articles satisfied all inclusion criteria at full-text review and were retained for data extraction. A list of exclusion reasons of references at the full-text stage is presented in Table A4.

**Table A4:** Exclusion criteria applied at full-text review.

Exclusion Criteria	Number of Records
1. Not English or no abstract available	1
2. Population clearly not adult humans	0
3. Population not related to inflammatory bowel disease	0
4. Intervention clearly non-intravenous	0
5. Outcomes not related to iron deficiency or hypophosphatemia	4
6. Outcomes not related to epidemiology or severity	14
7. Hypophosphatemia reported but no usable metrics	1
8. Not labeled as systematic review/meta-analysis	24
9. Duplicate record	4

Following the execution of the primary search strategies in PubMed and EMBASE, we conducted a brief, targeted supplementary search to minimize the risk of missing recently published or poorly indexed studies. Supplementary searches were conducted in a structured but pragmatic manner. Specifically, Google Scholar and specialist journal searches used the terms ‘iron deficiency anemia’ or ‘hypophosphatemia’ combined with keywords related to ‘inflammatory bowel disease’ and ‘intravenous iron’, and the ‘related articles’ function plus reference lists of included studies were screened. Through this process, two additional records were retrieved. In total, 14 studies (12 from databases and 2 from additional sources) form the evidence base for this review. Figure 4 in the main text presents the PRISMA flow diagram for this review.

#### Appendix B.3.6. Quality Assessment

As this umbrella review aimed to map the evidence landscape rather than evaluate the strength of evidence, and since many included systematic reviews/meta-analyses performed their own quality assessment, we did not use formal quality appraisal tools such as AMSTAR-2. Instead, we transparently reported the study design, sample size, and key methodological features in the Results section (Section 3), which allows readers to gauge the robustness of the evidence.
